# Integrated Transcriptomic and Proteomic Analyses Uncover the Regulatory Mechanisms of *Myricaria laxiflora* Under Flooding Stress

**DOI:** 10.3389/fpls.2022.924490

**Published:** 2022-06-10

**Authors:** Linbao Li, Guiyun Huang, Weibo Xiang, Haofei Zhu, Haibo Zhang, Jun Zhang, Zehong Ding, Jihong Liu, Di Wu

**Affiliations:** ^1^Rare Plants Research Institute of Yangtze River, China Three Gorges Corporation, Yichang, China; ^2^National Engineering Research Center of Eco-Environment Protection for Yangtze River Economic Belt, Beijing, China; ^3^Sanya Research Institute of Chinese Academy of Tropical Agricultural Sciences, Sanya, China; ^4^College of Horticulture and Forestry, Huazhong Agricultural University, Wuhan, China

**Keywords:** *Myricaria laxiflora*, post-flooding recovery, transcriptome and proteome, flooding stress, transcriptional and post-transcriptional regulation

## Abstract

Flooding is one of the major environmental stresses that severely influence plant survival and development. However, the regulatory mechanisms underlying flooding stress remain largely unknown in *Myricaria laxiflora*, an endangered plant mainly distributed in the flood zone of the Yangtze River, China. In this work, transcriptome and proteome were performed in parallel in roots of *M. laxiflora* during nine time-points under the flooding and post-flooding recovery treatments. Overall, highly dynamic and stage-specific expression profiles of genes/proteins were observed during flooding and post-flooding recovery treatment. Genes related to auxin, cell wall, calcium signaling, and MAP kinase signaling were greatly down-regulated exclusively at the transcriptomic level during the early stages of flooding. Glycolysis and major CHO metabolism genes, which were regulated at the transcriptomic and/or proteomic levels with low expression correlations, mainly functioned during the late stages of flooding. Genes involved in reactive oxygen species (ROS) scavenging, mitochondrial metabolism, and development were also regulated exclusively at the transcriptomic level, but their expression levels were highly up-regulated upon post-flooding recovery. Moreover, the comprehensive expression profiles of genes/proteins related to redox, hormones, and transcriptional factors were also investigated. Finally, the regulatory networks of *M. laxiflora* in response to flooding and post-flooding recovery were discussed. The findings deepen our understanding of the molecular mechanisms of flooding stress and shed light on the genes and pathways for the preservation of *M. laxiflora* and other endangered plants in the flood zone.

## Introduction

Flooding (including submergence and waterlogging) is a major problem that seriously influences plant growth and development worldwide, leading to significant loss of yield and even death of the plant (necrosis) (Jia et al., 2021). The frequency of flooding is increasing on natural and agricultural species by the building of dams and roads and divergence of rivers (Dat et al., 2004; Boulange et al., 2021). Under flooding environments, the access of plants to light and oxygen is drastically limited, accompanied by outbursts of reactive oxygen species (ROS). Meantime, the plant growth is suppressed, and the energy/nutrient supply is markedly inhibited (Yin and Komatsu, 2017).

Hormones have been demonstrated to play significant roles in flooding stress (Jia et al., 2021). Among them, ethylene is a primary signaling molecule and it is accumulated in plants to adapt to flooding stress (Kuroha et al., 2018). Abscisic acid (ABA), auxin, gibberellic acid (GA), cytokinin, and salicylic acid (SA) are other hormones that prevent plants against flooding stress by regulating adventitious root formation or by controlling carbohydrate consumption (Agullo-Anton et al., 2014; Du et al., 2015; Jia et al., 2021). Moreover, ethylene can interact with a hormonal cascade of ABA, GA, and auxin to promote adventitious root growth upon flooding in rice, bitterzoet, and tomato (Steffens et al., 2006; Vidoz et al., 2010; Dawood et al., 2016), supporting a complex regulation of hormones during flooding stress.

In addition to hormones, ROS plays vital roles in plant responses to flooding stress (Sasidharan et al., 2018). As an oxidative stress indicator, hydrogen peroxide is required for ethylene-induced epidermal impairments to promote aerenchyma formation under flooding conditions (Steffens and Sauter, 2009; Steffens et al., 2011). The activities of several ROS-scavenging enzymes, including catalase (CAT), peroxidase (POD), glutathione reductase (GR), and superoxide dismutase (SOD), are increased during the flooding stress (Ye et al., 2015; Wang et al., 2019). Meantime, ROS accumulation triggers the expression of downstream fermentation genes required for hypoxia (insufficient oxygen supply) acclimation and survival (Baxter-Burrell et al., 2002).

Transcription factors are also involved in the flooding responses and interact with hormones. For instance, *AtMYB2* and *AtbZIP50* are both induced by exogenous ABA, and their expression levels are increased under hypoxia conditions (Klok et al., 2002; Hsu et al., 2011; De Ollas et al., 2021). Two ethylene-responsive factors, *ERF1* and *ERF2*, are induced during the post-flooding recovery stage (Tsai et al., 2014; Yin et al., 2019). Overexpression of a NAC transcription factor (*SHYG*) enhances flooding-induced leaf movement and cell expansion in abaxial cells of the basal petiole region (Rauf et al., 2013). Two WRKY transcription factors, *WRKY33* and *WRKY12*, interact with each other to up-regulate *RAP2.2* for *Arabidopsis* adaptation to submergence stress (a state that the plant partially or fully immerses in water) (Tang et al., 2021). Meantime, WRKY-mediated transcriptional regulation triggers plant immunity upon flooding (Hsu et al., 2013).

Over the past decades, significant progress has been achieved regarding the plant response to flooding stress, and thousands of genes and many signaling pathways have been identified. A time-course transcriptomic analysis revealed that jasmonic acid (JA) participated in submergence-mediated internode elongation and improved flooding tolerance in rice (Minami et al., 2018). Meantime, the expression of genes referred to cell wall modification, trehalose biosynthesis, GA biosynthesis, and transcription factors was significantly changed (Minami et al., 2018). Analysis of enrichment pathways exhibited the involvement of antioxidant process, ROS signaling, photosynthesis, carbohydrate metabolism, stress response, hormone biosynthesis, and signal transduction in flooding stress (Wu et al., 2017; Wang et al., 2019; Qiao et al., 2020). Genes related to alcohol fermentation, ethylene biosynthesis, pathogen defense, and cell wall were altered at both transcriptional and proteomic levels, while ROS scavengers and chaperons were changed only at the translational level (Komatsu et al., 2009). Proteins involved in fermentation, ROS removal, glycolysis, and defense response were also affected under flooding treatments (Hashiguchi et al., 2009; Nanjo et al., 2012). These studies provided effective bases for exploring the mechanism of flooding responses at the transcriptomic and/or proteomic levels.

*Myricaria laxiflora* (Tamaricaceae) is an endangered plant mainly distributed in the flood zone of the Yangtze River, China (Li et al., 2021). In the native habitats, *M. laxiflora* is completely submerged during the long-lasting flooding periods in summer and rapidly sprouts in the autumn and winter after the flooding (Wu et al., 1998; Wang et al., 2003). Under summer flooding conditions, the primary and secondary branch number, aboveground biomass, and total biomass were greatly reduced in *M. laxiflora* seedlings (Chen and Xie, 2009). Meanwhile, the total soluble sugar, fructose, and starch contents also decreased in the branches and leaves (Zhou et al., 2021). Now summer flooding is considered as a major ecological process to influence the growth and development of *M. laxiflora*. In recent years, the construction of the Three Gorges Dam (TGD) significantly disrupted the *M. laxiflora* habitats, and only a few natural populations are remained (Bao et al., 2010). *M. laxiflora* has been regarded as an ideal plant to study the ecological restoration of Yangtze River (Chen and Xie, 2009; Chen et al., 2019). To date, many studies have been performed to investigate the influences of flooding on the seedling survival and physiology responses of *M. laxiflora* (Chen and Xie, 2009; Guan et al., 2020; Zhou et al., 2020); however, the molecular mechanisms underlying the flooding stress remain elusive.

In this work, we performed an integrated transcriptomic and proteomic analysis to investigate the dynamic changes in gene and protein expression of *M. laxiflora* during nine time-points under the flooding and post-flooding recovery treatments. These results shed light on the molecular mechanism of flooding stress in *M. laxiflora* and other endangered plants in the flood zone.

## Materials and Methods

### Plant Material and Experiment Design

The cuttings of a *M. laxiflora* plant were collected from Yanzhiba (Yichang, China) to prepare sufficient amount of experimental materials with the same genetic background. These materials were cultivated in the experimental farm of the Rare Plants Research Institute of Yangtze River in March 2020.

For the flooding treatment, 1-year-old seedlings with uniform growth were transferred to an experimental pond located in the TGD. The water in the experimental pond is connected to the Yangtze River, as it is a good strategy to simulate the natural flooding on *M. laxiflora*. The daily temperature ranged from 19 to 27°C. The flooding treatment started at 11:00 AM. The root samples were collected at 0, 6, 12, 18, 24, 30, 36, and 48 h (designated as R0, R6, R12, R18, R24, R30, R36, and R48) after the flooding treatment, respectively. Then, the seedlings were transferred to the normal condition for 12 h recovery (RR12), at which the root samples were also collected. The samples were immediately frozen in liquid nitrogen and stored at −80°C until use.

### Library Construction and Transcriptome Analysis

Total RNA extraction, library construction, and RNA-seq sequencing were performed by the Shanghai OE Biotech Company (Shanghai, China). Total RNA was isolated by the mirVana miRNA Isolation Kit (Ambion) according to the manufacturer’s protocol. RNA integrity was examined by the Agilent 2100 Bioanalyzer (Agilent Technologies, CA, United States). The RNA-seq libraries were constructed by the TruSeq Stranded mRNA LTSample Prep Kit (Illumina, CA, United States) and then sequenced on the Illumina HiSeq 2500 platform in PE150 model. Each sample was performed in triple replicates.

The clean reads were obtained by Trimmomatic (Bolger et al., 2014) to remove adaptors and low-quality reads. The transcripts were *de novo* assembled by Trinity (Grabherr et al., 2011) in paired-end method. The longest transcript was selected as a unigene according to the similarity and length for further analysis. Gene expression was calculated by using the FPKM (Fragments Per Kilobase per Million mapped reads) method.

### Proteomic Analysis

The samples used for RNA-seq sequencing were also subjected to proteome analysis by the Shanghai OE Biotech Company (Shanghai, China) using the data-independent acquisition (DIA) approach, which is regarded as a novel mass spectrometric method that shows higher precision and better reproducibility across replicates (Bruderer et al., 2015). Briefly, total protein was acquired from each sample, and the concentration was measured by the bicin-chonininc acid method. The quality was examined by SDS-PAGE with G250. After trypsin digestion, the peptides were desalted using a SOLA™ SPE 96 Column and then eluted with 60% methanol three times.

The LC–MS/MS assays were performed as previously described (Wang et al., 2020). Briefly, the peptide mixture was loaded onto an Agilent ZORBAX Extend-C18 reversed-phase column (5 μm, 15 cm × 2.1 mm) on an 1100 HPLC System (Agilent, CA, United States). Buffer A (2% ACN) and buffer B (90% ACN) were applied for the reverse gradient. The solvent gradient was set as follows: 0–10 min, 2% B; 10–10.01 min, 2–5% B; 10.01–37 min, 5–20% B; 37–48 min, 20–40% B; 48–48.01 min, 40–90% B; 48.01–58 min, 90% B; 58–58.01 min, 90–2% B; 58.01–63 min, 2% B. The flow rate was 250 μl/min. In total, 10 fractions were collected and then dried by vacuum centrifugation. For DIA mass spectrum scanning, dry gas was operated at 3.0 L/min at 180°C and the acquisition of MS data was performed in the mass range of 100–1700 m/z. The ion mobility was 0.7–1.3, and the collision energy was 20–59 eV.

The LC–MS/MS raw files were searched against the protein database derived from our RNA-seq transcripts by Spectronaut Pulsar software. The false discovery rate of proteins and peptides was set to 0.01. For any set of samples, the proteins with expression value in more than 50% samples were kept, and the missing value was filled with the mean in the same group.

### Transcriptomic and Proteomic Integrative Analysis

As previously described (Ding et al., 2020), the abundances of genes and proteins were min-max normalized between −1 and 1 among the time-points during flooding treatment. The expression patterns were identified by the standard procedure of WGCNA software (Langfelder and Horvath, 2008) and then visualized by the R package pheatmap. For further analysis, genes and proteins that were not assigned to any of the groups were excluded.

To interpret the biological functions influenced by flooding treatment, the genes were functionally annotated and classified into Gene Ontology (GO) categories by Blast2GO (Conesa et al., 2005), or assigned into hierarchical categories by the MapMan classification system (Thimm et al., 2004). The significantly enriched categories were determined by the Fisher’s exact test (Ding et al., 2020).

### Statistical Analysis

Differentially expressed genes (DEGs) were identified by DESeq2 (Love et al., 2014) setting the false discovery rate < 0.05 and |log_2_FC (fold-change)| > 1. Differentially expressed proteins (DEPs) were identified by the Student’s *t*-test setting a fold-change > 2 (or <0.5) and *P*-value < 0.05. Each sample was performed in triple replicates. The correlations between the DEPs and their corresponding genes were calculated by the Pearson’s correlation test (Ding et al., 2019).

## Results

### Differentially Expressed Genes and Differentially Expressed Proteins Identification

Significantly altered phenotypes were observed in *M. laxiflora* as the flooding treatment time progressed: the color of the leaves was changed from bright green to yellow–green and small aerial roots began to grow. To investigate the dynamic expression changes of genes and proteins, root samples were collected at 0, 6, 12, 18, 24, 30, 36, and 48 h after flooding treatment and at 12 h after post-flooding recovery treatment, and subjected to transcriptome and proteome analyses, respectively.

**FIGURE 1:**
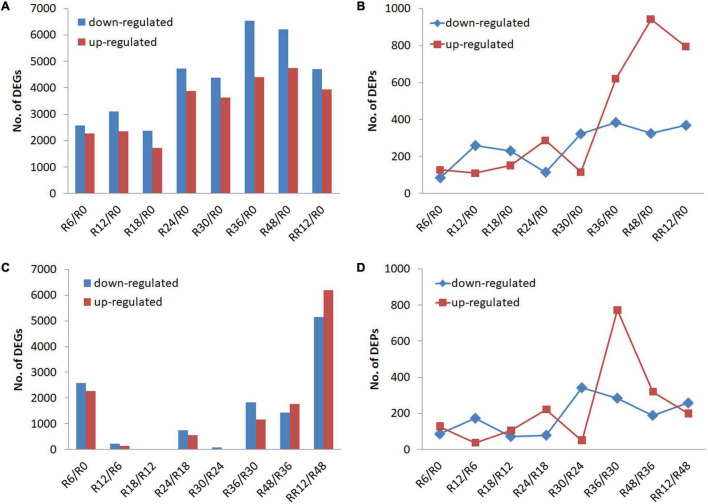
Transcriptome and proteome profiling of *M. laxiflora* at different time-points during flooding and post-flooding recovery treatment. **(A,B)** Up-regulated and down-regulated DEGs and DEPs, respectively, identified for a certain period of flooding compared with the control (R0). **(C,D)** Up-regulated and down-regulated DEGs and DEPs, respectively, identified between a time point and the preceding time point. R0, R6, R12, R18, R24, R30, R36, and R48 represent the samples collected at 0, 6, 12, 18, 24, 30, 36, and 48 h after the flooding treatment, respectively, while RR12 indicates the samples collected at 12 h after post-flooding recovery treatment.

Differentially expressed genes and DEPs were identified between a time point and R0 (0 h, the control) to signify the genes and proteins up-regulated or down-regulated for a certain period of flooding (Figures 1A,B). The number of DEGs and DEPs fluctuated slightly and then increased as the flooding stress time prolonged, although there was a time-shift (e.g., R24 vs. R36) between the transcriptomic and proteomic levels.

Differentially expressed genes and DEPs were also identified between a time point and the preceding time point to signify significant transitions in gene and protein expression during flooding stress. The highest number of DEGs was detected from R48 to RR12 (RR12/R48), followed by R6/R0, while less number of DEGs was found at R12/R6, R18/R12, and R30/R24 (Figure 1C). On the contrary, the highest number of up-regulated and down-regulated DEPs was detected at R36/R30 and R30/R24, respectively (Figure 1D).

Subsequently, these DEGs and DEPs were merged, and their co-expression patterns were detected at the transcriptomic and/or proteomic levels to dissect their over-represented functions, respectively.

### Differentially Expressed Genes Exclusively Identified at the Transcriptome Level

**FIGURE 2:**
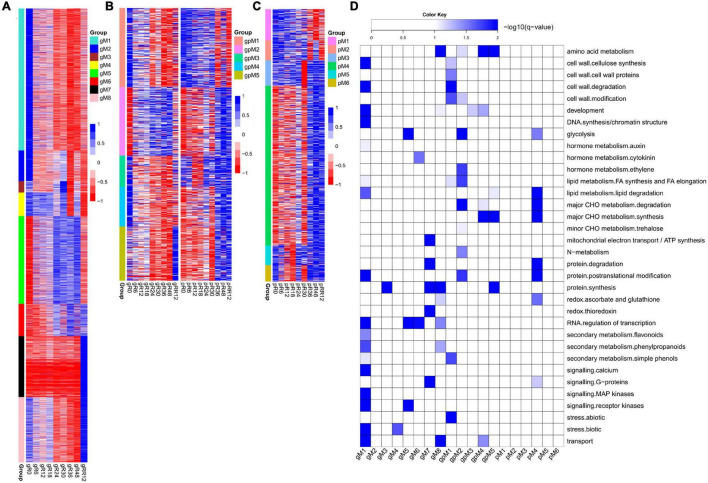
Dynamic and stage-specific expression changes of genes and proteins upon flooding stress. **(A)** Profiles of DEGs identified exclusively at the transcriptomic level. A total of eight groups (gM1–gM8) were determined according to their expression patterns. **(B)** Profiles of DEGs/DEPs identified at both the transcriptomic and proteomic levels. Five groups (gpM1–gpM5) were found. **(C)** Profiles of DEPs identified exclusively at the proteomic level. Six groups (pM1–pM6) were found. **(D)** Functional category enrichment of DEGs/DEPs corresponding to those identified in **(A–C)**. The samples/groups are prefixed as follows: g, transcriptomic level; p, proteomic level; gp, both transcriptomic and proteomic levels.

In total, eight groups (gM1–gM8) of DEGs were determined at the transcriptome level but not at the proteome level (Figure 2A and Supplementary Table 1).

Genes from gM1 to gM3 showed the highest expression at R0 and their levels were dramatically depressed upon flooding treatment. The genes from gM1 were significantly enriched in auxin metabolism, cell wall, RNA regulation of transcription, secondary metabolism, calcium signaling, and MAP kinase signaling (Figure 2D). Many genes included in this group were involved in cellulose synthesis (*CESA6*, *CSLC4*, *CSLC6*, *CSLD3*, *CSLD5*), cell wall degradation (*XYL4*, *ASD1*, *BXL1*, *BXL2*), and lignin biosynthesis (*4CL2*, *4CL3*, *COMT1*, *CCOAMT*, *CCR1*, *CAD5*, *CAD9*). In addition, many auxin-responsive genes (*SAUR39*, *SAUR55*, *DFL2*, *TCP14*) and auxin efflux carriers (*PIN1*, *PIN4*, *PIN7*, *LAX3*) were also included. These results strongly suggested a coordinated contribution of auxin, cell wall, and lignin biosynthesis genes to growth inhibition under flooding treatment. Accordingly, several genes referred to calcium signaling (*CAM8*, *CBL10*, *CML37*, *CML41*, *CPK1*, *CPK13*), MAP kinase signaling (*MAPKKK5*, *MAPKKK13*, *MKK3*–*MKK7*, *MPK15*, *MPK16*), and abiotic stress (*DIL9*, *RD22*, and *ERD3* for drought; *HSFA2* and *HSP90.1* for heat) were identified, supporting a major role of these genes in rapid response to flooding stress. However, no enrichment was found for gM2 and gM3.

Genes from gM4 maintained a high expression level from R0 to R18 and then fluctuated until RR12. These genes were significantly enriched in stress response, of which *CSC1*, *CTL1*, and *RBOHE* were involved in drought, heat, and defense responses.

Genes from gM5 to gM6 were highly expressed from R24 to R48, although their levels were quite different at RR12. The enriched categories of gM5 were glycolysis, RNA regulation of transcription, and receptor kinase signaling. Many glycolysis-related genes (*GAPDH1*, *ENO1*, *PFK2*, *PFK3*, *PGK*, *PGM2*, *TPI*) were found in this group. A few receptor kinases related to abiotic stress (*CRK29* and *RLK7*) and root hair elongation (*MRH1*) were also found. The genes from gM6 were enriched in cytokinin metabolism and RNA regulation of transcription. A cytokinin receptor (*HK3*) and two cytokinin synthesis-degradation genes (*IPT3* and *CKX1*) were included in this group. The results indicated a significant role of these genes in the late stages (e.g., R24–R48) of flooding stress.

Genes from gM7 to gM8 were expressed highest at RR12. The enriched categories of gM7 were mitochondrial electron transport/ATP synthesis, protein synthesis and degradation, thioredoxin (TRX) redox, and G-protein signaling. Several genes referred to mitochondrial electron transport/ATP synthesis (*ATP3*, *ATP5*, *COB*, *PHB1*, *PHB7*) and redox reaction (*CAT2*, *CSD2*, *GPX7*, *GR1*, *PER1*, *TRX*) were included in this group. The genes from gM8 were enriched in development, redox, RNA regulation of transcription, and secondary metabolism. Many genes involved in meristem development (*AP2* and *NAC2*), dormancy (*EMB2750* and *EMB2746*), plant growth (*CPL4*, *AGL8*, *EMF2*), and lignin biosynthesis (*C4H*, *4CL*, *COMT1*, *CCoAOMT1*, *CCR1*) were included. In addition, a few genes involved in ascorbate biosynthesis (*GME*) and ROS scavenging (*APX1*, *FSD3*, *CSD1*) were also found in this group. These results indicated a demand of energy for plant growth at the recovery stage after flooding.

Together, these findings revealed stage-specific functions of genes exclusively regulated at the transcriptome level. For example, auxin, cell wall, calcium signaling, and MAP kinase signaling were associated with the early stage of flooding stress; glycolysis, cytokinin, and RNA regulation of transcription were related to the late stage; while mitochondrial electron transport/ATP synthesis, redox, and lignin biosynthesis were associated with the recovery stage after flooding.

### Differentially Expressed Genes/Differentially Expressed Proteins Identified at the Transcriptome and Proteome Levels

Five groups of genes (gpM1–gpM5) were determined to be differentially expressed at both the transcriptome and proteome levels (Figure 2B and Supplementary Table 1).

The genes from gpM1 (*R* = 0.72, *P* = 0.028) to gpM2 (*R* = 0.57, *P* = 0.113) exhibited similar expression trends between the transcriptome and proteome levels. The expression levels of gpM1 were gradually deceased from R0 to RR12, and the genes of this group were significantly enriched in cell wall, FA synthesis and elongation, simple phenols, and abiotic stress (Figure 2D). Many cell wall related genes, including *CSLA2*, *IRX1*, and *IRX6* for cellulose synthesis, *FLA8* and *FLA17* for cell wall proteins, *GH9B5* and *BXL2* for cell wall degradation, and *EXLA1*, *EXLB1*, and *EXPA4* for cell wall modification were included. Similarly, two simple phenol genes (*LAC12* and *LAC17*) involved in oxidation reduction and lignin catabolic processes, and a few genes referred to salt (*OSM34*), cold (*RCI3* and *ESK1*), and heat response (*HSP18.2* and *HSP17.3*) were also included.

In contrast to gpM1, the expression of genes in gpM2 was gradually increased from R0 to RR12. These genes were significantly enriched in glycolysis, ethylene, major CHO metabolism, N-metabolism, and protein posttranslational modification (Figure 2D). Many genes related to glycolysis (*GAPDH3*, *PFK2*, *PFK3*, *PFP1*, *PFP2*, *PK1*, *PK2*) and major CHO degradation (*SEX1*, *HXK1*, *SUS3*, *SUS4*) were found in this group. In addition, a few genes referred to trehalose biosynthesis (*TPS6* and *TPS7*), N-metabolism (*GLT1* and *NIA1*), ethylene metabolism (*ERS1* and *ACO1*), and abiotic stress (*TAP46*, *SnRK2*, and *OST1*) were also included. These results strongly suggested a request for energy generation *via* carbohydrate degradation and glycolysis pathways to defend against the flooding stress.

The genes from gpM4 exhibited an opposite expression trend (*R* = −0.72, *P* = 0.029) between the transcriptome and proteome profiles. The enriched categories were amino acid (AA) metabolism, development, major CHO metabolism, and transport. Several genes involved in starch synthesis (*APL1*, *SBE2.2*, and *SS4*) and degradation (*GWD3* and *PHS1*), cell proliferation and growth (*ANT*, *LUG*, *RAPTOR1*), and AA metabolism (*FAH*, *ADT1*, *URE*, *PGDH2*) were found in this group. Interestingly, three transporters (*CUE1*, *PHT1;1*, *PDR2*) involved in phosphate transport were included.

However, no correlation was detected between the transcriptome and proteome levels for the genes from gpM3 to gpM5. The genes from gpM3 were enriched in development, and a few genes referred to cell division (*SWA1* and *ELO1*), apical meristem development (*NAC2*), and leaf senescence (*DET1* and *YLS8*) were included. The enriched categories of gpM5 were AA metabolism, lipid degradation, synthesis of major CHO metabolism, and protein synthesis. Several genes involved in AA biosynthesis (*MS1*, *HMT2*, *P5CS1*) and starch synthesis (*APL3*, *ISA1*, *GBSS1*) were found in this group.

Collectively, these results suggested a crucial contribution of transcriptional regulation (e.g., in cell wall and abiotic stress) and post-transcriptional regulation (e.g., in AA metabolism, development, and major CHO metabolism) upon flooding stress.

### Differentially Expressed Proteins Exclusively Identified at the Proteome Level

Six groups (pM1–pM6) of DEPs were determined at the proteome level but not at the transcriptome level (Figure 2C and Supplementary Table 1).

The expression of proteins in pM4 was gradually increased from R0 to RR12. These proteins were enriched in glycolysis, major CHO metabolism, redox, and G-proteins signaling (Figure 2D). Many proteins referred to glycolysis (ENO3, PPC2, PPC3, PFK3, PK), major CHO synthesis (APL2, SBE2.2, SS1, SS3, SPS1F, SPS2F), and degradation (DPE1, DPE2, ISA3, SEX4, AMY3, BMY3, PHS2) were included. In addition, a few proteins related to redox (APX3, MDAR6, OXP1, GPX8, TO1, TY1) were also found in this group.

The proteins from pM1 to pM3 were highly expressed from R0 to R24, while those from pM5 to pM6 were highly expressed from R24 to RR12. However, no functional enrichment was significantly determined for these groups.

**FIGURE 3:**
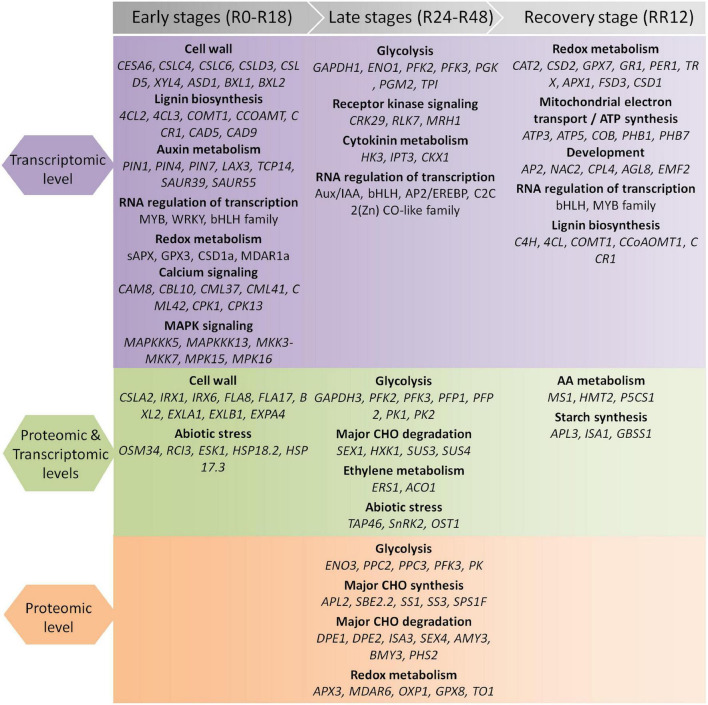
Summary of genes and pathways identified by integrated transcriptomic and proteomic analysis during flooding stress. The flooding treatment is divided into the early, late, and recovery stages.

Together, these findings revealed stage-specific expression patterns of genes exclusively regulated at the protein level. The key genes and pathways determined by our integrated transcriptome and proteome analysis were summarized in Figure 3.

### Response of Genes Involved in Sucrose-Starch Metabolism and Glycolysis

**FIGURE 4:**
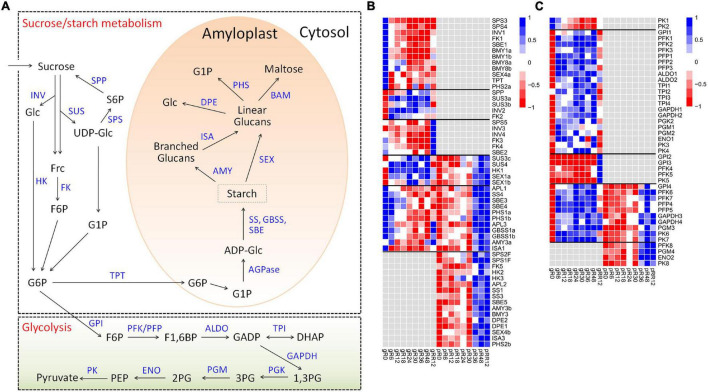
Expression profiles of DEGs and DEPs related to sucrose-starch metabolism and glycolysis during flooding stress. **(A)** Summary of pathways of sucrose-starch metabolism and glycolysis. **(B)** Heatmap of DEGs and DEPs related to sucrose-starch metabolism. **(C)** Heatmap of DEGs and DEPs involved in glycolysis metabolism. The samples are prefixed as follows: g, transcriptomic level; p, proteomic level.

Diverse expression patterns were observed for the sucrose-starch metabolism genes at the transcriptome and/or proteome levels (Figures 4A,B and Supplementary Table 2).

Many genes involved in sucrose degradation (*INV1*, *FK1*) and starch degradation (*BMY1a*, *BMY1b*, *BMY8a*, *BMY8b*, *SEX4a*, *PHS2a*) were down-regulated at R6 by flooding at the transcriptome level. Several starch biosynthesis genes (including *APL1*, *APL3*, *SS4*, *SBE3*, *SBE4*, *GBSS1a*, *GBSS1b*) were also down-regulated, but they showed ∼6 h delay compared with those degradation genes, indicating a quicker response of starch degradation genes than the biosynthesis genes in response to flooding.

A few sucrose degradation genes were up-regulated at R6–R48 by flooding (*SUS3a*, *SUS3b*, *INV2*, *FK2*) or at RR12 by recovery treatment (*INV3*, *INV4*, *FK3*, *FK4*) exclusively at the transcriptome level. By comparison, many starch degradation genes (*AMY3b*, *BMY3*, *DPE1*, *DPE2*, *SEX4b*, *ISA3*, *PHS2b*) were up-regulated at R36–RR12 exclusively at the proteome level, suggesting different regulation mechanisms of sucrose-starch genes under flooding treatment. In addition, more than a dozen genes related to sucrose degradation (*SUS3c*, *SUS4*, *HK1*), starch biosynthesis (*APL1*, *APL3*, *SS4*, *SBE3*, *SBE4*, *GBSS1*), and starch degradation (*SEX1a*, *SEX1b*, *PHS1a*, *PHS1b*, *AMY3a*, *ISA1*) were regulated at both the transcriptome and proteome levels with low expression correlations, supporting a major contribution of post-transcriptional regulation in sucrose-starch metabolism under flooding.

Similar expression patterns were found for the glycolysis genes (Figures 4A,C), since most of them were up-regulated at distinct stages during flooding at the transcriptome, proteome, or both levels. Notably, 21 genes covering the whole glycolysis pathway were up-regulated from R6 to R48 at the transcriptome level, suggesting an emergency of energy demand under flooding treatment.

Collectively, these results clearly suggested stage-specific responses of sucrose-starch and glycolysis genes under flooding treatment mainly *via* post-transcriptional regulation.

### Response of Genes Associated With Redox Metabolism

**FIGURE 5:**
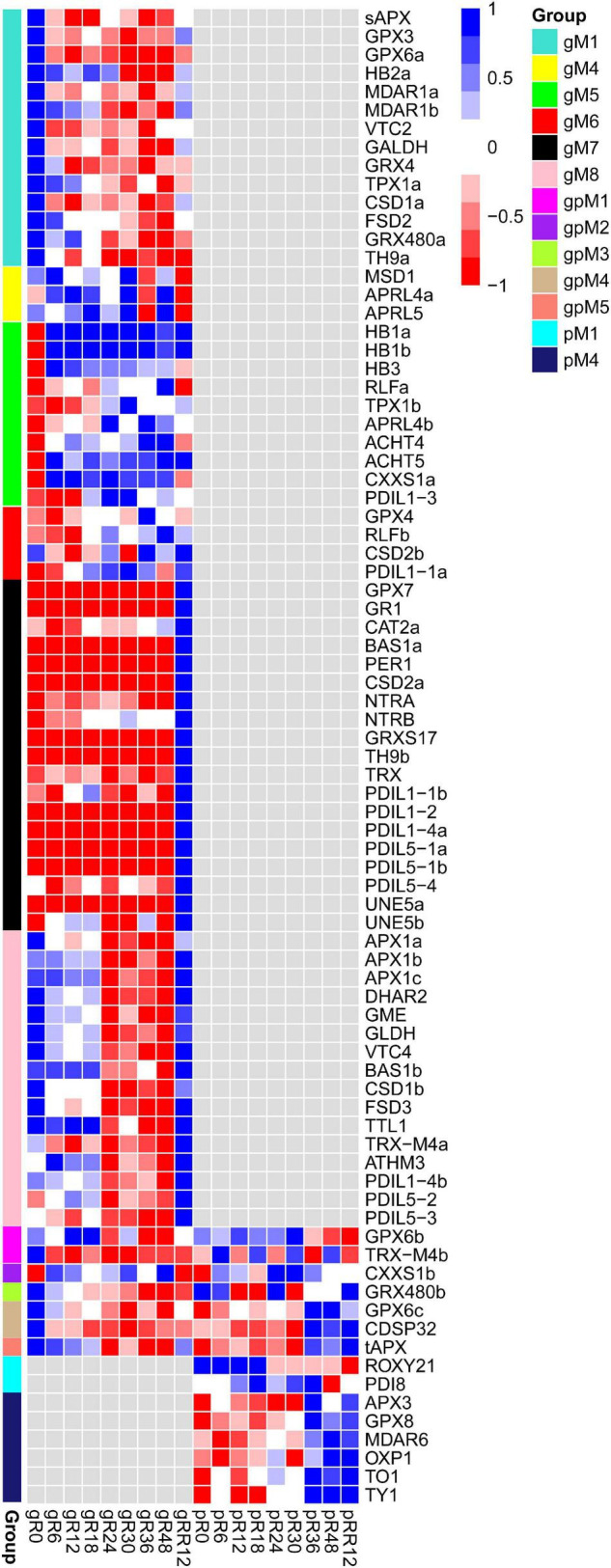
Expression profiles of DEGs and DEPs involved in redox metabolism during flooding stress. Each row represents a gene with its name at the right.

In total, 81 redox genes were differentially expressed in response to flooding, with the four most abundant types were TRX, SOD, glutathione peroxidase (GPX), and ascorbate peroxidase (APX). These genes were mainly grouped into gM1, gM5, gM7, gM8, and pM4 (Figure 5 and Supplementary Table 3).

Many genes involved in ascorbate biosynthesis (*VTC2*, *GALDH*), hormone response (*HB2a*, *GPX3*, *GRX480a*), abiotic stress (*sAPX*, *GPX6a*, *FSD2*, *CSD1a*), and ROS removal (*MDAR1a*, *MDAR1b*) were found in gM1, and their expression levels were significantly depressed by flooding stress. In comparison, several genes related to cold stress (*HB1a*, *HB1b*) and lateral root development (*RLFa*, *RLFb*) were included in gM5, and they were up-regulated under flooding treatment. The genes in gM7 and gM8 were significantly up-regulated at RR12, indicating their major roles at the recovery stage. A few genes involved in temperature stress (*BAS1*, *GRXS17*) and superoxide detoxification (*CAT2a*, *CSD2a*) were found in gM7, while those related to H_2_O_2_ scavenger (*APX1a*), ascorbate biosynthesis (*GME*, *GLDH*, *VTC4*), and abiotic stress (*CSD1b*) were found in gM8. In addition, several genes involved in cold stress (*MDAR6*) and oxidative damage (*APX3*, *GPX8*) were found in pM4. These redox genes were regulated only at the transcriptome or proteome level.

On the contrary, a few genes functioned in drought (*CDSP32*), oxidative stress (*GPX6b* and *GPX6c*), and ROS-scavenging (*tAPX* and *GRX480b*) were regulated both at the transcriptome and proteome levels.

Together, these results suggested that redox genes were coordinately regulated in a stage-specific manner at the transcriptome and/or proteome levels under flooding treatment.

### Response of Genes Involved in Hormone Metabolism

**FIGURE 6:**
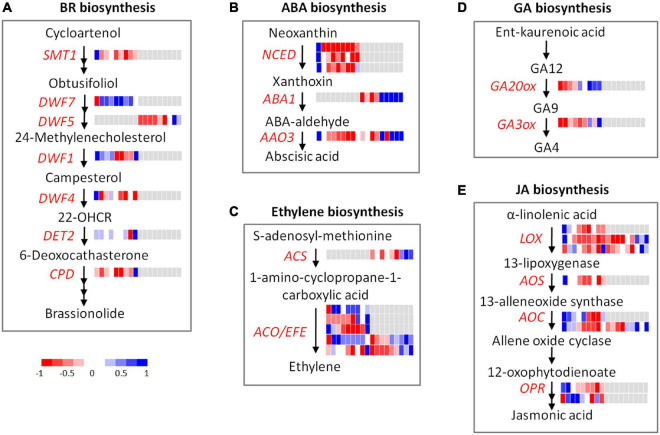
Expression profiles of DEGs and DEPs involved in hormone metabolism during flooding stress. **(A)** BR biosynthesis, **(B)** ABA biosynthesis, **(C)** ethylene biosynthesis, **(D)** GA biosynthesis, **(E)** JA biosynthesis. Each row represents a gene, and the cells (from left to right) indicate the normalized expression value of R0, R6, R12, R18, R24, R30, R36, R48, and RR12 at the transcriptomic and proteomic levels, respectively.

Diverse stage-specific expression patterns were observed for the genes involved in hormone biosynthesis and signaling transduction (Figure 6 and Supplementary Table 4).

Four auxin influx carriers (*LAX3*, *PIN1*, *PIN4*, *PIN7*) and three auxin-responsive genes (*DFL2*, *SAUR39*, *TCP14*) were highly expressed at R0, and their expression levels were greatly depressed by flooding treatment (Supplementary Table 4). Two auxin-responsive genes (*ARF19*, *PID*) were highly expressed from R0 to R18, while two auxin receptors (*TIR1*, *AFB3*) were highly expressed from R24 to R48 at the transcriptome level. *PIN3* showed similar expression patterns to *TIR1* and *AFB3* at the transcriptome level, but it was also regulated at the proteome level. An auxin transport (*BIG*) was up-regulated from R36 to RR12 exclusively at the proteome level.

Likely, the brassinosteroid (BR) biosynthesis and signaling genes were highly expressed at R0 (*SMT1*, *DWF4*, *BIN2*, *BEH4a*, *BEH4b*), R6–R36 (*DWF7* and *BZR1*), and RR12 (*CPD*, *TRIP1*, *DET2*, and *DWF1*) at the transcriptome level, respectively. In addition, *DWF5* was highly expressed at R48–RR12 exclusively at the proteome level.

Four ABA biosynthesis genes (*NCED3b*, *NCED4*, *NCED3a*, *AAO3*) and a few ABA-responsive genes (*ABI1*, *CCD8*, *HVA22J*) were highly expressed at R0 and their expression levels were down-regulated by flooding stress. Interestingly, *NCED3a* and *AAO3* were up-regulated from R48 to RR12 while *NCED3b* and *NCED4* were not changed, indicating their different roles at the recovery stage. In addition, *AAO3* and *ABA1* were also up-regulated from R36 to RR12 at the proteome level.

Seven genes involved in cytokinin biosynthesis-degradation (*IPT1a*, *IPT1b*, *IPT3a*, *CKX5*) and signal transduction (*HK5a*, *WOLa*, *WOLb*) were highly expressed at R0, and their expression was greatly depressed by flooding treatment. By contrast, another six genes involved in cytokinin biosynthesis-degradation (*IPT3b*, *CKX1*) and signal transduction (*HK1*, *HK3a*, *HK3b*, *HK3c*) were highly induced from R24 to RR12.

Five genes involved in ethylene biosynthesis (*EFEa*, *EFEb*) and signal transduction (*ERF4*, *ETR2b*, *EIN4*) were highly expressed at RR12 at the transcriptome level. On the contrary, another six genes related to ethylene biosynthesis (*ACO1a*, *ACO1b*) and signal transduction (*ERF3b*, *ETR1*, *ETR2a*, *ERS1*) were highly expressed from R6 to R48. Moreover, *ERS1* and *ACO1b*, together with three ethylene biosynthesis genes (*EFEc*, *EFEd*, *ACS10*), showed high expression levels from R36 to RR12 at the proteome level.

*GA20OX1* and *GA3OX1* were two vital genes involved in GA biosynthesis. These two genes and a GA receptor (*GID1B*) were specially induced at RR12 at the transcriptome level.

Four JA biosynthesis genes (*OPR3*, *AOS*, *LOX2*, *AOC3*) were highly expressed at R0–R12, while *OPR1* was highly expressed from R6 to R18 at the transcriptome level. *AOC4*, *LOX3*, and *LOX5* were highly expressed from R0 to R12 at the transcriptome level and from R36 to RR12 at the proteome level.

Collectively, these results revealed diverse stage-specific expression patterns of hormone genes during flooding treatment: ABA and JA mainly functioned at the early stage, GA mainly functioned at the recovery stage, while auxin, BR, CK, and ethylene functioned across all stages. These results also suggested a major role of hormone genes in flooding stress *via* post-transcriptional regulation.

### Response of Transcriptional Factors

**FIGURE 7:**
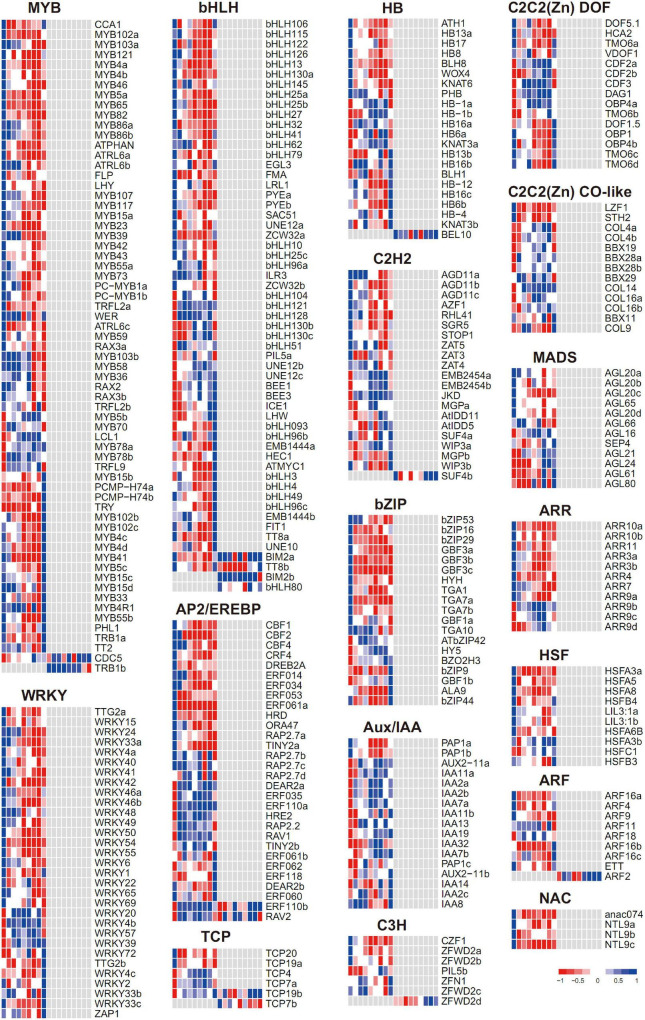
Expression profiles of transcriptional factors during flooding stress. Each row represents a TF with its name at the right. The cells (from left to right) indicate the normalized expression value of R0, R6, R12, R18, R24, R30, R36, R48, and RR12 at the transcriptomic and proteomic levels, respectively.

In total, 349 differentially expressed transcriptional factors (TFs) were found, with most of them regulated exclusively at the transcriptome level (Figure 7 and Supplementary Table 5). The TF families with the most abundant numbers were MYB (65) and bHLH (57), and then followed by WRKY (31), AP2/EREBP (30), HB (22), and C2H2 (21). Notably, the TFs from each family trended to be enriched in a few expression patterns (e.g., gM1 and gM5), indicating the stage-specific roles of TFs under flooding treatment. Since the TFs were overall enriched in gM1, gM5, and gM8, these three groups were further inspected to interpret their molecular functions, respectively.

In gM1, many stress-responsive TFs were found from diverse gene families, including AP2/EREBP (*CBF1*, *CBF2*, *DREB2A*), bZIP (*GBF3a-c*), C2H2 (*AZF1*, *RHL41*), and MYB (*MYB65*) for low temperature, AP2/EREBP (*DREB2A*, *ERF53*) and MYB (*MYB102a*) for drought, and AP2/EREBP (*HRD*), C2H2 (*AZF1*), and WRKY (*WRKY33a*) for salt. Hormones play an important role in stress response, and accordingly, AP2/EREBP (*CBF1*, *CBF2*) and bZIP (*GBF3a-c*) for ABA, ARF (*ARF4*, *ARF16a*) for auxin, ARR (*ARR3a-b*, *ARR4*, *ARR10*, *ARR11*) for cytokinin, and HB (*ATH1*) for GA responses were found. Meantime, bHLH (*bHLH32*, *LRL1*) for root hair development, bHLH (*EGL3*) and MYB (*MYB5a*, *MYB23*) for trichome development, and MYB (*FLP*) and bHLH (*FMA*) for stomata development were observed. Interestingly, a few TF families involved in nutrition response were also included, including bHLH (*PYEa-b*) for iron response, C2H2 (*STOP1*) for aluminum toxicity, and WRKY (*WRKY6*) and bHLH (*bHLH32*) for phosphorus response.

In gM5, the members from AP2/EREBP (*RAV1*) and C2C2(Zn) CO-like (*BBX29*) related to low temperature, C2C2(Zn) DOF (*DAG1*) involved in seed germination, and MYB (*MYB5b*) responsible for trichome development were found.

In gM8, the members from bHLH (*TT8a*) and MYB (*MYB5c*) for trichome development, HB (*HB6b*) and MYB (*MYB102b-c* and *MYB41*) for abiotic stress and ABA response, bHLH (*FIT1*) for iron uptake, and MYB (*TT2*) and bHLH (*TT8a*) for anthocyanin biosynthesis were included.

These results suggested stage-specific roles of TFs under flooding treatment: e.g., TFs related to abiotic stress, hormone, development, and nutrition were depressed at the early-stage (gM1), those involved in cold and seed germination were highly expressed at the mid-stage (gM5), while those referred to development, abiotic stress, and anthocyanin biosynthesis were induced at the recovery-stage (gM8).

## Discussion

### The Genes Regulated Both at the Transcriptomic and Proteomic Levels

Transcriptome and proteome methods have widely been applied to explore DEGs and DEPs in response to flooding stress (Nanjo et al., 2012; Minami et al., 2018). However, very few studies were performed by integrative analysis of these two approaches, a promising way to systematically investigate complex physiological processes (Ding et al., 2020). To better understand the molecular mechanisms underlying flooding response, transcriptome and proteome were carried out in parallel to identify the critical genes and proteins under a naturally simulated flooding stress in *M. laxiflora* in this work. In total, 16,893 DEGs and 1,900 DEPs were identified under flooding. Of which, only 955 were commonly found at the transcriptome and proteome levels (Supplementary Table 1), suggesting that the proteins with significant expression changes did not always show a corresponding change at the transcriptome level.

Many genes and pathways have been demonstrated to be involved in flooding responses (Nanjo et al., 2012; Minami et al., 2018); however, their regulatory roles remain elusive at both the transcriptome and proteome levels. Carbohydrate metabolism genes were significantly depressed by flooding at the transcriptome level (Chen et al., 2016). This phenomenon was also observed in this work. Besides, a few sucrose degradation genes were up-regulated by flooding (*SUS3a*, *SUS3b*, *INV2*, *FK2*) or by post-flooding recovery treatment (*INV3*, *INV4*, *FK3*, *FK4*) exclusively at the transcriptome level (Figure 4B). In addition to the starch degradation genes (*AMY3b*, *BMY3*, *DPE1*, *DPE2*, *SEX4b*, *ISA3*, *PHS2b*) up-regulated by flooding exclusively at the proteome level, many starch biosynthesis (*APL1*, *APL3*, *SS4*, *SBE3*, *SBE4*, *GBSS1*) and degradation (*SEX1a*, *SEX1b*, *PHS1a*, *PHS1b*, *AMY3a*, *ISA1*) genes were regulated at both the transcriptome and proteome levels with low expression correlations (Figure 4B). These results supported a complex regulation of carbohydrate metabolism genes under flooding conditions (Wang and Komatsu, 2018).

Due to the energy shortage instigated by reduced carbohydrate metabolism, glycolysis is activated to produce energy for plant survival under flooding conditions (Lin et al., 2019). Consistently, diverse regulatory patterns were observed for the glycolysis genes at transcriptomic and/or proteomic levels (Figure 4C), in accord with the dynamic changes of starch-sucrose genes. Proteins related to AA metabolism, transport, and development were identified in soybean in response to flooding stress (Yin et al., 2014). Similarly, many genes relevant to AA metabolism (*FAH*, *ADT1*, *URE*, *PGDH2*), phosphate transport (*CUE1*, *PHT1;1*, *PDR2*), and apical meristem development (*NAC2*, *DET1*, *YLS8*) were identified in this work. Moreover, these genes displayed negatively correlations or no correlations between the transcriptomic and proteomic levels (Supplementary Table 1).

On the contrary, many genes related to cell wall (including *CSLA2* and *IRX1* for cellulose synthesis, *GH9B5* and *BXL2* for cell wall degradation, *EXLA1*, *EXLB1*, and *EXPA4* for cell wall modification) and abiotic stress (*OSM34*, *RCI3*, *ESK1*, *HSP18.2*), which also played an essential role in flooding response (Komatsu et al., 2009; Minami et al., 2018), showed a consistent expression change at the transcriptome and proteome levels, indicating a major role of transcriptional regulation in flooding response.

These results deepen our understanding of the regulatory mechanisms of diverse gene pathways and suggested a major contribution of both transcriptional and post-transcriptional regulation to flooding stress.

### The Regulatory Network of *Myricaria laxiflora* Under Flooding

**FIGURE 8:**
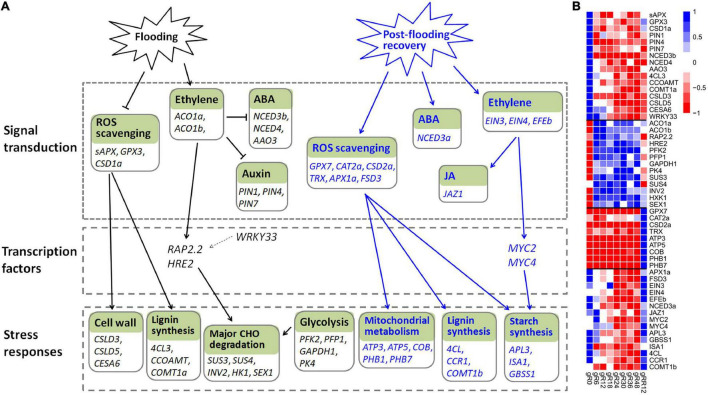
The transcriptional regulation model of *M. laxiflora* during flooding stress. **(A)** The transcriptional regulatory networks of *M. laxiflora* upon flooding (black lines) and post-flooding recovery (blue lines) treatment. The normal arrows and T-bars indicate positive and negative regulation relationships, respectively. Solid lines represent the relationships that are consistent with previously reported studies, while dash lines represent the relationships reported in other plants but not confirmed in our work. **(B)** The transcriptomic profiles of genes present in **(A)**.

Ethylene is a primary signal to trigger plant-adaptive responses to flooding (Kuroha et al., 2018). It accumulates quickly under flooding conditions, and the activities of ethylene biosynthetic enzymes (such as ACO and ACS) are induced in the meantime (Van Veen et al., 2013; Sasidharan and Voesenek, 2015). The elevated ethylene causes a massive depression of ABA, mediated by the down-regulation of 9-*cis*-epoxycarotenoid dioxygenase and the up-regulation of ABA-8-hydroxylase (Van Veen et al., 2013). In lines with previous studies, two ACO genes (*ACO1a* and *ACO1b*) were induced by flooding, while three genes (*NCED3b*, *NCED4*, *AAO3*) involved in ABA biosynthesis were greatly down-regulated in this work. Meantime, ethylene inhibited auxin transport in roots and hampered the normal growth of adventitious roots (Vidoz et al., 2010), and accordingly, the expression levels of three auxin transporters (*PIN1*, *PIN4*, *PIN7*) were depressed (Figure 8 and Supplementary Table 6).

Reactive oxygen species is another important signal in hypoxia sensing and ethylene-mediated root development (Steffens et al., 2011). Consistently, a few ROS scavenging genes (*sAPX*, *GPX3*, *CSD1a*) were depressed by flooding stress. Several genes related to calcium signaling (*CAM8*, *CBL42*, *CML37*, *CPK1*) and MAPK signaling (*MAPKKK5*, *MKK3*, *MPK15*), which are key signals involved in flooding and abiotic stress (Zeng et al., 2017; Wang and Komatsu, 2018), were also dramatically depressed.

ERF transcription factors play an essential role in flooding and hypoxic stress (Van Dongen and Licausi, 2015). *RAP2.2* and *HRE2*, which were involved in hypoxia-responsive gene regulation and flooding survival (Sasidharan and Voesenek, 2015), were significantly up-regulated by flooding in this work. *RAP2.2* also induced the expression of genes encoding sugar metabolism (Hinz et al., 2010). However, the transcription factor *WRKY33*, which up-regulated *RAP2.2* for *Arabidopsis* adaptation to submergence stress (Tang et al., 2021), was down-regulated by flooding, indicating a different regulatory network of *M. laxiflora* upon flooding.

Flooding-induced reduction in oxygen and CO_2_ inhibits photosynthesis and aerobic respiration and causes a strong decline in energy and carbohydrate availability (Yin and Komatsu, 2017). Consistent with the severely reduction in cell wall formation (Bailey-Serres and Voesenek, 2010), a few cell wall (*CSLD3*, *CSLD5*, *CESA6*) and lignin biosynthesis (*4CL3*, *COMT1a*, *CCOAMT*) genes were down-regulated by flooding. To cope with energy supply, the sucrose-starch and glycolysis pathways are activated (Bailey-Serres and Voesenek, 2008). Accordingly, several genes involved in glycolysis (*PFK2*, *PFP1*, *GAPDH1*, *PK4*) and major CHO metabolism (*SUS3*, *SUS4*, *INV2*, *HK1*, *SEX1*) were greatly induced (Figure 8). Collectively, these results provide a complex regulatory network of *M. laxiflora* under flooding stress.

### The Regulatory Network of *Myricaria laxiflora* Upon Post-flooding Recovery

Flooding responses have been extensively studied in plants, however, less is known about the signaling networks at the post-flooding recovery stage (Yeung et al., 2018).

Reactive oxygen species acts as a primary signaling in plant post-flooding recovery due to the combined effects of reoxygenation, reillumination, dehydration, and senescence stresses (Yeung et al., 2019). ROS generated during post-flooding disrupts the photosynthetic apparatus, and consequently, hampers the photosynthetic recovery and carbohydrate replenishment (Sasidharan et al., 2018). Therefore, the ROS scavengers are subsequently activated during recovery (Yeung et al., 2018). In this work, many genes related to ROS scavenging (*GPX7*, *CAT2a*, *CSD2a*, *TRX*, *APX1a*, *FSD3*) were up-regulated at post-flooding recovery stage. This efficient ROS scavenging may result in faster starch replenishment, correlating with higher biomass and survival ratios (Qin et al., 2013; Yeung et al., 2018). Accordingly, many genes related to starch biosynthesis (*APL3*, *ISA1*, *GBSS1*), development (*AP2*, *NAC2*, *EMB2746*, *AGL8*), and lignin biosynthesis (*4CL*, *CCR1*, *COMT1b*) were up-regulated upon post-flooding recovery (Figure 8 and Supplementary Table 6). ROS accumulation caused by reoxygenation and reillumination is also associated with the high-energy demands of reactivated mitochondrial metabolism (Sasidharan et al., 2018), therefore, a few genes referred to mitochondrial electron transport/ATP synthesis (*ATP3*, *ATP5*, *COB*, *PHB1*, *PHB7*) were up-regulated by post-flooding recovery treatment (Figure 8). These results suggested that the quickly growth of *M. laxiflora* during post-flooding recovery might due to its highly efficient ROS scavenging system (Yeung et al., 2018).

In addition to ROS, hormones and TFs also confer post-flooding recovery responses (Yeung et al., 2019). Several ethylene biosynthesis genes (*ACS2*, *ACS8*, *ACO2*) and ethylene-responsive factors (*ERF1* and *ERF2*) were induced upon reoxygenation (Tsai et al., 2014). Over-expression of *MYC2* enhanced stress tolerance to reoxygenation by JA signaling during submergence recovery (Yuan et al., 2017). Furthermore, *MYC2* physically interacted with the transcription factors *EIN3* and *EIL1* to regulate ethylene-mediated post-flooding responses (Song et al., 2014). In this work, an ethylene-forming enzyme (*EFEb*) and two ethylene receptors (*EIN3* and *EIN4*) were greatly induced by post-flooding recovery treatment. However, none of the ethylene-responsive factors (*ERF1*, *ERF3*, and *ERF4*) showed a similar expression pattern, indicating distinct regulatory pathways of ethylene in post-flooding recovery between *M. laxiflora* and *Arabidopsis* (Song et al., 2014).

*MYC2* and *MYC4* function redundantly in JA signaling (Song et al., 2014). These two genes, together with their interaction component *JAZ1* involved in JA signaling (Zhou et al., 2016), were significantly up-regulated upon recovery. *MYC2* also co-expressed with several starch synthesis genes (*APL3*, *ISA1*, and *GBSS1*) during recovery, suggesting a possible involvement of *MYC2* in carbohydrate metabolism *via* regulating the expression of starch-metabolic genes (Bian et al., 2021). In addition, *NCED3a*, a key gene involved in ABA biosynthesis, was significantly induced upon recovery, in accord with the crucial roles of ABA in post-flooding recovery (Yeung et al., 2018). Together, these results suggested a complex regulatory network of ROS, JA, ethylene, and ABA signaling in *M. laxiflora* upon post-flooding recovery.

## Conclusion

In summary, the molecular mechanisms underlying flooding stress were investigated by integrated transcriptomic and proteomic approaches in *M. laxiflora* in nine time-points. Our results not only uncovered the highly dynamic and stage-specific expression changes of genes/proteins during flooding and post-flooding recovery, but also highlighted the genes and pathways that functioned specially at different stages. The genes involved in auxin, cell wall, calcium signaling, and MAP kinase signaling were depressed during the early stages of flooding at the transcriptomic level, the genes referred to glycolysis and major CHO metabolism mainly functioned during the late stages of flooding at the transcriptomic and/or proteomic levels, while those genes related to ROS scavenging, mitochondrial metabolism, and development were activated upon post-flooding recovery at the transcriptomic level. In addition, the genes/proteins related to redox, hormones, and TFs also played vital roles in flooding stress of *M. laxiflora*. The findings will improve our understanding of molecular mechanisms of flooding stress, and provide useful information for the preservation of *M. laxiflora* and other endangered plants in the flood zone.

## Data Availability Statement

The original contributions presented in the study are publicly available. The accession numbers are here: NCBI, PRJNA840865 and iProX, IPX0004477000.

## Author Contributions

LL, DW, and JL conceived and designed the research and revised the manuscript. GH, WX, HFZ, HBZ, and JZ performed the experiments. LL and ZD analyzed the data and drafted the manuscript. All authors contributed to the article and approved the submitted version.

## Conflict of Interest

LL, GH, HFZ, HBZ, JZ, and DW were employed by the China Three Gorges Corporation. The remaining authors declare that the research was conducted in the absence of any commercial or financial relationships that could be construed as a potential conflict of interest.

## Publisher’s Note

All claims expressed in this article are solely those of the authors and do not necessarily represent those of their affiliated organizations, or those of the publisher, the editors and the reviewers. Any product that may be evaluated in this article, or claim that may be made by its manufacturer, is not guaranteed or endorsed by the publisher.
